# [Retracted] Upregulation of miR-34a by diallyl disulfide suppresses invasion and induces apoptosis in SGC-7901 cells through inhibition of the PI3K/Akt signaling pathway

**DOI:** 10.3892/ol.2025.15111

**Published:** 2025-05-27

**Authors:** Guojun Wang, Guanghui Liu, Yanwei Ye, Yang Fu, Xiefu Zhang

Oncol Lett 11: 2661–2667, 2018; DOI: 10.3892/ol.2016.4266

Following the publication of the above article, a concerned reader drew to the Editor's attention that there were a large number of potential anomalies associated with the Transwell cell assay data shown in Fig. 2A-E on p. 2664; essentially, groupings of cells appeared to be markedly similar in appearance both looking within, and comparing across, the five data panels in these figures.

After having conducted an internal investigation of the data in this paper, the Editor of *Oncology Letters* has judged that the potentially anomalous presentation of the strikingly similar groupings of cells in Fig. 2 was too extensive that these features could have been attributed to pure coincidence. Therefore, the Editor has decided that this article should be retracted from the publication on the grounds of an overall lack of confidence in the data. The authors were asked for an explanation to account for these concerns, but the Editorial Office did not receive a reply. The Editor sincerely apologizes to the readership for any incovenience caused, and we thank the reader for bringing this matter to our attention.

